# The Story of the Insane from Year to Year

**Published:** 1893-02-11

**Authors:** 


### THE STORY OF THE INSANE FROM YEAR TO YEAR.

(Fifth Series.)

### SUSSEX COUNTY ASYLUM, HAYWARDS HEATH.

The thirty-third annual report shows that the number of patients had during the year 1891 decreased from 844 to 792; but this was in great part owing to the transfer of patients from the asylum to workhouses or to other asylums. The admissions were 306, the recoveries 82, and the deaths 116. The recoveries were 24·2 per cent. on the admissions, and the deaths were 14·5 on the average number resident. Hereditary tendency and drink together account for 73 of the admissions, and consumption was the cause of death in 17 cases. Post-mortem examinations were made in 52 instances. Dr. Saunders says, and we fully agree with him, that the feelings of the patients' friends ought so be respected in these things, and that these examinations should not be made when the friends are opposed to them. When an asylum boasts that a *post-mortem* is made in every case of death there is something wrong somewhere. There is a balance of £440 in favour of ths farm, which balance seems to have been obtained in the same way as that of the year 1890. The weekly rate of maintenance was 9s. 10¼d. per head. The head attendant, Mr. Buckle, retired during the year, and we are pleased to learn he received the full amount of pension allowed by the statute. It is to be hoped he will long live to enjoy his well-earned income.

### THE BOROUGH ASYLUM, LEICESTER.

At the end of 1891 this asylum completed the twenty-second year of its existence and published its twenty-second annual report. The numbers rose from 477 to 517. There were 104 admissions, 36 recoveries, and 22 deaths. After setting on one side the transfers, the percentages of recoveries comes out at the satisfactory rate of 45, and the death-rate was only 4·37. This being far below the average in other asylums, and also below the average of this asylum in former years. There were only three deaths from consumption. Influenza visited the asylums, and 40 men and six women were attacked. For the previous year these numbers were almost reversed, there being 40 women and four men stricken with the disease. There does not seem to have been any death from it. The Visiting Committee say they "have pleasure in again recording their appreciation of the highly satisfactory manner in which Dr. Finch and the officers generally have discharged their respective duties." The cost per head per week was 8s. 8d.

### NORWICH CITY ASYLUM.

This is situated at Hellesdon, near Norwich, and it now produces its twelfth annual report. It contained, Dr. Harris tells us, "at midnight between December 31st, 1890, and January 1st, 1891," 259 patients, and this number had risen during the year to 283 patients. The year's admissions amounted to 103, the recoveries to 35, and the deaths to 25. Eighteen of the admissions were caused by drink, and 28 by hereditary tendency. The percentage of recoveries on admissions is 38·88, and the percentage of deaths on the average number resident is 6·90. Ambulance lectures were commenced, but, we are told, did not prove successful, and so instruction has been given at opportune occasions in the wards, and the "results have been more advantageous to all concerned." Dr. Harris's experience seems to have been somewhat exceptional, and asylum reports generally show that these classes are gladly welcomed by the attendants and nurses. We do not envy Dr. Harris's experience with his staff, for we find that his assistant medical officer resigned in June, and his successor left in December to be succeeded by Dr. Sykes, who now holds the appointment. The Commissioners say that "one strong dress and one pair of black eyes were all the indications we saw of any turbulent or destructive habits." The Visiting Committee note "the continued excellence of service rendered by our Medical Superintendent, ensuring as it does the comfort of the patients, the confidence of your committee, and the high standing of our asylum among the city and borough asylums of the kingdom." The weekly rate of maintenance was 9s. 1⅝d. per head.

### THE LAWN, LINCOLN.

This is a small registered hospital, containing on the last day of 1891 only 61 patients. The admissions numbered 15, the recoveries 9, and the deaths 3. The percentage of recoveries on the admissions stands at 64·3, and is the highest recovery rate we have yet come to. The deaths were only 5 per cent. on the average number resident. The Medical Superintendent does not publish a report; but, indeed, in such a small asylum it would be hardly necessary, and it would be well-nigh impossible to find something new to say every year. The Governors "express their satisfaction with the manner in which Dr. Russell has conducted the arrangements of the institution, and appreciate the anxiety he has always shown for its welfare and that of the patients under his care." The Commissioners in Lunacy say "the accommodation here is decidedly good for the payments made, and we think that the hospital only requires to be wider known for the benefits to be more widely appreciated." We did not notice any table giving the scale of payments, so we cannot enlighten our readers on the point.

